# Exercise mediates the sex differences in adult visuospatial cognitive ability

**DOI:** 10.3389/fpsyg.2025.1629724

**Published:** 2025-08-13

**Authors:** Ruiqi Ruan, Junsheng Hong, Yan Huang, Fan Yuan, Xuehao Zhang, Yefei Mo, Tianxiao Hu, Yuhe Liu

**Affiliations:** ^1^School of Medical Technology and Information Engineering, Zhejiang Chinese Medical University, Hangzhou, China; ^2^Department of Otolaryngology, Head and Neck Surgery, Beijing Friendship Hospital, Capital Medical University, Beijing, China; ^3^Department of Radiology, Beijing Friendship Hospital, Capital Medical University, Beijing, China; ^4^Department of Otolaryngology, Head and Neck Surgery, Sir Run Run Shaw Hospital, Zhejiang University, Zhejiang, China; ^5^College of Life Science, Zhejiang Chinese Medical University, Hangzhou, China; ^6^Department of Endocrinology, The 903rd Hospital of PLA, Hangzhou, China

**Keywords:** exercise, gender differences, visuospatial cognition, mental rotation, spatial memory, spatial navigation

## Abstract

**Background:**

Sex differences in visuospatial cognitive performance have been consistently documented, with males typically demonstrating superior performance in tasks requiring spatial processing. While multiple biological, psychological, and sociocultural factors have been proposed to explain these differences, the role of physical exercise as a potential mediator remains understudied. Given that females typically engage in less physical exercise than males globally, this study aimed to investigate whether exercise intensity serves as a mediating factor in the observed sex/gender differences in visuospatial cognitive ability.

**Methods:**

A total of 224 undergraduate students (112 men and 112 women) aged 18–25 years participated in a comprehensive battery of visuospatial cognitive tests, including assessments of mental rotation, spatial memory, and spatial navigation ability. Participants’ weekly exercise patterns were assessed using the validated Godin-Shephard Leisure-Time Physical Activity Questionnaire, with activities categorized into strenuous, moderate, and mild exercise. Mediation analyses were conducted using the PROCESS macro (Model 4) with 5,000 bootstrap iterations, controlling for spatial anxiety, sense of direction, and childhood spatial activity experience.

**Results:**

Males significantly outperformed females across all visuospatial cognitive domains (all *p* < 0.05). Mediation analysis revealed that leisure time activity score significantly mediated sex/gender differences in visuospatial cognitive ability, with indirect effects ranging from 20 to 34% of the total effect. When examining exercise intensity separately, strenuous exercise emerged as the primary mediator, particularly in mental rotation (indirect effect = 0.073, 95% CI [0.021, 0.134]) and spatial memory tests (forward span: indirect effect = 0.073, 95% CI [0.017, 0.147]; backward span: indirect effect = 0.069, 95% CI [0.009, 0.143]). The mediating effect of strenuous exercise was more pronounced in tasks with higher cognitive demands.

**Conclusion:**

Physical exercise, particularly strenuous exercise, partially mediates sex differences in visuospatial cognitive abilities among young adults. These findings suggest that promoting increased participation in strenuous physical activities among women may help reduce gender disparities in visuospatial cognition. However, the cross-sectional nature of this study precludes causal inferences, and future longitudinal or intervention studies are needed to confirm these relationships.

## Introduction

### Background

Visuospatial cognitive ability encompasses the brain’s capacity to perceive, process, manipulate, and navigate spatial information in both two-and three-dimensional environments (Pinker, 1984). This cognitive domain includes several interrelated but distinct components: mental rotation (the ability to mentally manipulate objects), spatial memory (encoding and retrieval of spatial information), and spatial navigation (wayfinding and orientation in space). These abilities are fundamental for numerous daily activities and academic pursuits, from interpreting maps and assembling furniture to excelling in science, technology, engineering, and mathematics (STEM) fields (Alvarez-Vargas et al., 2020).

Sex differences in visuospatial abilities represent one of the most robust findings in cognitive psychology, with meta-analyses consistently demonstrating male advantages, particularly in mental rotation tasks (Voyer et al., 1995). While these differences are well-documented, their etiology remains multifaceted and debated. Current theoretical frameworks propose an interplay of biological, psychological, and sociocultural factors. Biological explanations include sex hormone influences on brain development and function (Ingalhalikar et al., 2014), as well as structural differences in neural connectivity patterns (Bettio et al., 2020). Psychological factors encompass spatial anxiety and self-confidence disparities (Alvarez-Vargas et al., 2020; Arrighi and Hausmann, 2022), while sociocultural influences include gender-stereotyped toy preferences and activity patterns during childhood.

An emerging area of interest concerns the role of physical exercise in cognitive function. Accumulating evidence suggests that regular physical activity enhances various cognitive domains through multiple neurobiological mechanisms, including increased neuroplasticity, improved cerebrovascular function, and enhanced neurotrophic factor expression (Rogge et al., 2017). Specifically for visuospatial abilities, balance training has been shown to improve spatial cognition in healthy adults, while professional athletes demonstrate superior mental rotation performance compared to non-athletes (Weigelt and Memmert, 2021). However, the literature also reveals inconsistencies, with some studies finding limited or domain-specific effects of exercise on cognition (Madden et al., 1989; Hötting et al., 2012).

Critically, a substantial gender gap exists in physical activity participation globally. Recent epidemiological data indicate that 31.7% of women exhibit insufficient physical activity levels compared to 23.4% of men (The Lancet Public H, 2019), with this disparity widening across the lifespan (Nowak et al., 2019). This systematic difference in exercise engagement between genders raises an important but understudied question: Could differential exercise patterns contribute to the observed sex differences in visuospatial cognitive abilities?

Previous research examining exercise effects on visuospatial cognition has been limited by several methodological constraints. Many studies focused on single gender samples to avoid confounding effects (Jansen et al., 2012; Graydon, 1980), thereby precluding direct examination of exercise as a mediator of sex differences. Additionally, most investigations have concentrated on older adults or clinical populations, with limited data on healthy young adults. Furthermore, the role of exercise intensity—a critical parameter in exercise-cognition research—remains largely unexplored in the context of sex differences in visuospatial abilities.

To ensure a rigorous examination of exercise’s mediating role, we selected covariates based on three key theoretical considerations. First, spatial anxiety was included because heightened anxiety during spatial tasks can impair performance by consuming cognitive resources and triggering avoidance behaviors. Second, sense of direction was controlled as it reflects an individual’s self-efficacy and habitual strategies in spatial processing, which may independently influence task performance. Third, childhood spatial activities were accounted for given their demonstrated long-term effects on neural development and spatial skill acquisition. These covariates collectively address the psychological (anxiety), metacognitive (self-assessment), and developmental (early experience) dimensions that could confound the relationship between exercise and visuospatial abilities. By controlling for these potential confounders, our design aims to more precisely isolate the role of exercise intensity in mediating sex differences—addressing critical gaps in the existing literature.

Specifically, the present study targets key limitations identified earlier: the understudied role of exercise as a mediator of sex differences in visuospatial cognition, the lack of focus on exercise intensity, and the overreliance on non-young adult or single-gender samples. Thus, we address these gaps by examining whether and how exercise intensity mediates sex differences in visuospatial cognitive abilities among healthy young adults. Building on theoretical frameworks of embodied cognition and neuroplasticity, we hypothesize that: (1) males will demonstrate superior performance across visuospatial tasks compared to females; (2) physical exercise, particularly strenuous exercise, will significantly mediate these sex differences; and (3) the mediating effect of exercise will be more pronounced in tasks requiring greater cognitive resources.

## Methods

### Participants

*A priori* power analysis using G*Power 3.1 indicated that a minimum sample size of 200 participants would be required to detect medium effect sizes (f^2^ = 0.15) in mediation analyses with 80% power at *α* = 0.05. A total of 236 participants (117 females and 119 males) were recruited from Zhejiang Chinese Medicine University, Zhejiang University, and Hangzhou Medical College between March 2023 and December 2023. Recruitment was conducted through campus advertisements and course announcements, with efforts to ensure balanced gender representation.

*Inclusion criteria were*: (1) age 18–25 years; (2) right-handedness as determined by the Edinburgh Handedness Inventory (laterality quotient > 40, 3) normal or corrected-to-normal vision; (Ingalhalikar et al., 2014) Montreal Cognitive Assessment (MoCA) score ≥ 26.

*Exclusion criteria included*: (1) history of neurological or psychiatric disorders; (2) current use of psychoactive medications; (3) history of head injury with loss of consciousness; (4) vestibular disorders, dizziness, or vertigo; (5) hearing impairment; (6) uncorrected visual impairments; (7) motor disabilities affecting computer use; (8) diagnosed attention deficit disorders. Of the 236 participants screened, 12 were excluded due to incomplete questionnaire responses (*n* = 6), failure to complete spatial tests (*n* = 5), or MoCA score below 26 (*n* = 1), resulting in a final sample of 224 participants (112 females, 112 males; mean age = 20.20 years, SD = 1.79).

### Procedure

The study was approved by the Ethical Committee of Peking University and Peking University First Hospital (Approval no. 2021-390). All procedures were conducted in accordance with the Declaration of Helsinki. Written informed consent was obtained from all participants prior to testing.

Testing sessions were conducted individually in a quiet laboratory setting and lasted approximately 60 min. The testing protocol consisted of: (1) demographic information collection and screening assessments; (2) physical activity assessment using the Godin-Shephard Leisure-Time Physical Activity Questionnaire; (3) visuospatial cognitive test battery administered via tablet device; (4) completion of covariate questionnaires (spatial anxiety, sense of direction, childhood spatial activities). All tasks were presented in a fixed order to minimize order effects across participants. Participants were offered breaks between tasks to prevent fatigue.

### Measures

#### Physical activity assessment

Physical activity was assessed using the Godin-Shephard Leisure-Time Physical Activity Questionnaire (GSLTPAQ) (Amireault and Godin, 2015; Godin, 2011), a validated self-report measure widely used in exercise-cognition research. Participants reported the frequency of engaging in strenuous (e.g., running, competitive sports), moderate (e.g., fast walking, easy bicycling), and mild (e.g., easy walking, yoga) exercise sessions lasting at least 15 min during a typical week. The Leisure Time Activity Score was calculated as: (9 × strenuous frequency) + (5 × moderate frequency), following established scoring procedures that exclude mild exercise due to its minimal metabolic impact (Amireault and Godin, 2015). This scoring method has demonstrated good test–retest reliability (r = 0.74) and concurrent validity with objective activity measures.

While self-report measures have limitations including potential overestimation of activity levels, the GSLTPAQ was selected for its brevity, established psychometric properties, and specific relevance to leisure-time activities that are more likely to reflect volitional exercise patterns.

#### Visuospatial cognitive assessment

**Figure 1:**
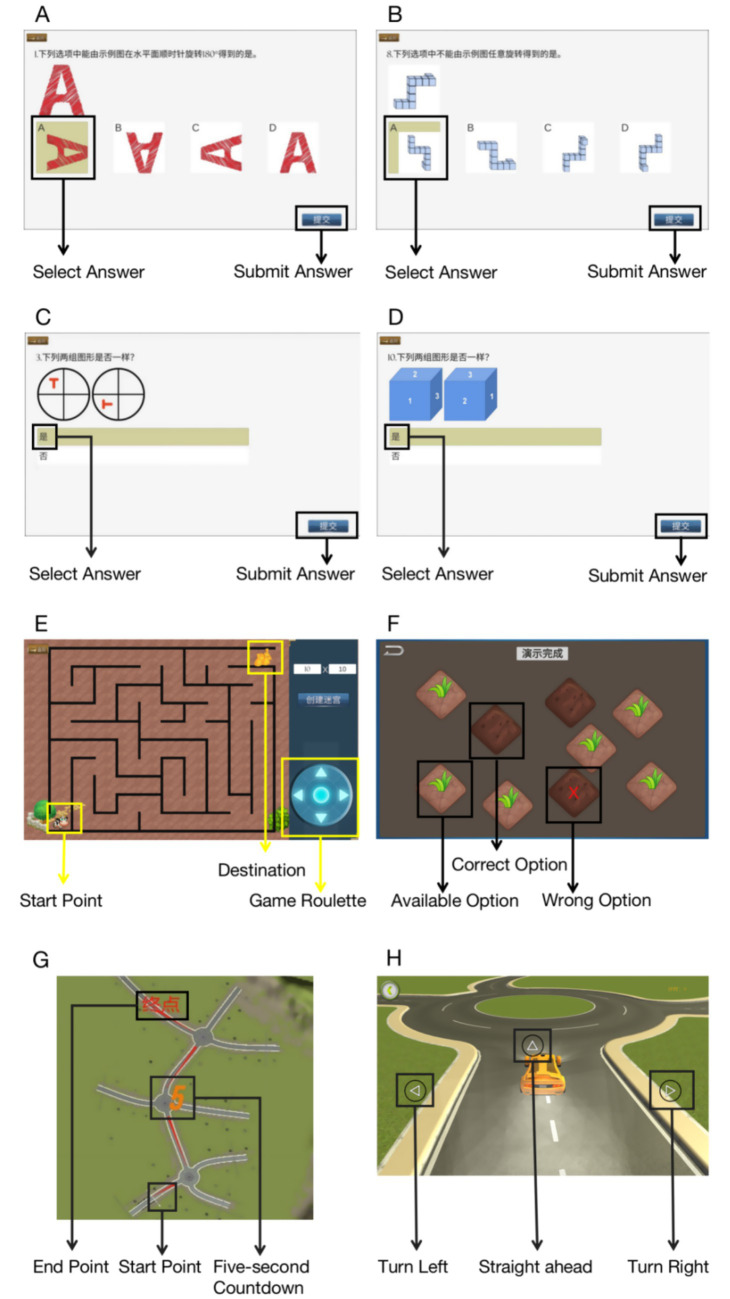
Visuospatial cognitive assessment system (VCAS) test examples. Test battery interface showing: **(A)** 2D object mental rotation (high difficulty) - participants identify which rotated figures match the reference; **(B)** 3D object mental rotation (high difficulty) - participants match 3D rotated objects; **(C)** 2D object comparison (low difficulty) - participants judge if two figures are identical after rotation; **(D)** 3D object comparison (low difficulty) - participants compare rotated 3D objects; **(E)** Maze navigation test (10 × 10 grid shown) - participants navigate from start to finish; **(F)** Spatial memory test (Weeding test) - participants reproduce sequences of highlighted squares; **(G)** 3D driving test map - participants memorize directional choices for three islands; **(H)** 3D driving test interface - participants navigate based on memory. All tests were administered on tablet devices with touch or keyboard input as appropriate.

Visuospatial abilities were assessed using the Visuospatial Cognitive Assessment System (VCAS) (Huang et al., 2023), a tablet-based test battery developed and validated by our research group. The VCAS demonstrates good psychometric properties and includes four tests targeting different aspects of visuospatial cognition (Figure 1):

1. *Card rotation test (mental rotation)*: this test assessed two-dimensional and three-dimensional mental rotation abilities across two difficulty levels. In the low difficulty condition (8 items), participants judged whether two figures were identical after rotation. In the high difficulty condition (10 items), participants compared four rotated options to a reference figure and identified matches or mismatches. Unlike traditional speeded tests, participants were given unlimited time to minimize the confounding effects of processing speed on sex differences (Alvarez-Vargas et al., 2020). Scores reflected accuracy (number correct).
2. *Spatial memory tests (weeding test)*: based on the Corsi Block paradigm, this test evaluated forward and backward spatial span. Nine squares appeared on screen, with sequences of 2–9 blocks (forward) or 2–8 blocks (backward) highlighted sequentially. Participants reproduced sequences in the same (forward) or reverse (backward) order. Performance metrics included: (a) memory span (longest sequence correctly recalled); (b) span product (span × number of correct trials); (c) clicking velocity (total clicks/total time). The span product was used as the primary outcome as it incorporates performance across all difficulty levels.
3. *Maze navigation test*: this test assessed spatial navigation ability using algorithmically generated maze maps at two complexity levels (10 × 10 and 18 × 18 grids). Participants navigated from the bottom-left to top-right corner using arrow keys, with only one correct path available. Performance was measured by completion time (seconds) and number of steps taken. Lower times indicated better performance.
4. *3D driving test*: this test combined spatial memory and navigation demands. Participants memorized a map showing three islands with directional choices, displayed for 5 s. They then navigated through the islands from memory, with incorrect choices requiring reselection. Performance metrics included thinking time (seconds) and selection errors (proportion of incorrect choices). Lower values indicated better performance.

### Covariates

Three theoretically relevant covariates were assessed to control for potential confounding factors:

1. *Spatial anxiety scale* (Lawton and Kallai, 2002): an 8-item measure assessing anxiety in spatial situations (*α* = 0.85 in current sample). Higher scores indicate greater spatial anxiety.
2. *Santa Barbara sense of direction scale* (Hegarty, and Dept of Psychology, Santa-Barbara, CA, US, hegarty@psych. ucsb.edu, Richardson AEUC-SB, Dept of Psychology, Santa-Barbara, CA, US, Montello DRUC-SB, Dept of Geography, Santa-Barbara, CA, US, montello@geog.ucsb.edu, Lovelace KUC-SB, Dept of Psychology, Santa-Barbara, CA, US, Subbiah IUC-SB, 2002): a 15-item self-report measure of environmental spatial ability (α = 0.88 in current sample). Higher scores indicate better self-perceived navigation ability.
3. *Childhood spatial activity questionnaire*: a 26-item inventory assessing engagement in spatial (14 items) and non-spatial (12 items) activities during ages 3–12. The ratio of spatial to total activities was calculated as the covariate.

### Statistical analyses

All analyses were conducted using SPSS 26.0 (IBM Corp., Armonk, NY). Prior to analysis, data were screened for outliers using z-scores (|z| > 3.29) and assumptions of normality (Shapiro–Wilk test), homogeneity of variance (Levene’s test), and multicollinearity (VIF < 5) were verified. Missing data (< 1%) were handled using listwise deletion.

Sex differences in visuospatial performance and physical activity were examined using independent samples *t*-tests for normally distributed variables and Mann–Whitney U tests for non-normal distributions. Effect sizes were calculated as Cohen’s d for *t*-tests and r for Mann–Whitney U tests.

Mediation analyses were conducted using the PROCESS macro v4.1 for SPSS (Model 4) with 5,000 bias-corrected bootstrap samples. Two sets of analyses were performed: (1) simple mediation with leisure time activity score as the mediator; (2) parallel mediation with strenuous, moderate, and mild exercise frequencies as simultaneous mediators. All models included spatial anxiety, sense of direction, and childhood spatial activity ratio as covariates. Sex was coded as 0 = male, 1 = female. Indirect effects were considered significant when 95% confidence intervals excluded zero.

To address multiple comparisons, we applied False Discovery Rate (FDR) correction using the Benjamini-Hochberg procedure with q = 0.05. Additionally, we conducted sensitivity analyses to examine the robustness of findings to different analytical decisions.

## Results

### Sample characteristics and sex differences

Table 1 presents descriptive statistics and sex comparisons for all study variables. The final sample of 224 participants was well-balanced by sex (112 males, 112 females) with comparable age distributions [males: M = 20.32, SD = 1.81; females: M = 20.08, SD = 1.77; *t*(222) = 1.01, *p* = 0.314].

**Table 1:** Descriptive statistics and sex comparisons for all study variables.

Variable	Male (*n* = 112)	Female (*n* = 112)	Test statistic	*p*-value	Effect size
Demographics					
Age (years), M (SD)	20.32 (1.81)	20.08 (1.77)	t = 1.01	0.314	d = 0.13
Visuospatial cognitive tests					
Card rotation (low), Mdn (IQR)	8 (7, 8)	7 (7, 8)	U = 5244.5	0.004**	r = 0.19
Card rotation (high), Mdn (IQR)	9 (8, 9)	7.5 (7, 9)	U = 4296.5	<0.001***	r = 0.34
Forward span product, Mdn (IQR)	406 (282, 544)	288 (247.50, 397.25)	U = 4290.0	<0.001***	r = 0.34
Forward span velocity, M (SD)	0.43 (0.07)	0.42 (0.06)	t = 1.16	0.249	d = 0.15
Backward span product, Mdn (IQR)	291 (252, 413)	258 (165, 350)	U = 4549.5	<0.001***	r = 0.31
Backward span velocity, Mdn (IQR)	0.43 (0.34, 0.52)	0.43 (0.30, 0.58)	U = 6176.5	0.899	r = 0.01
10 × 10 maze time (s), Mdn (IQR)	22 (17, 27)	26.5 (21, 37)	U = 4753.0	<0.001***	r = 0.30
10 × 10 maze steps, Mdn (IQR)	32 (26, 40)	34 (28, 46)	U = 5528.0	0.088	r = 0.11
18 × 18 maze time (s), Mdn (IQR)	64.5 (49, 94)	76.5 (59, 101)	U = 5075.5	0.002**	r = 0.21
18 × 18 maze steps, Mdn (IQR)	90 (72, 112)	86 (74, 120)	U = 6081.0	0.643	r = 0.03
3D driving time (s), Mdn (IQR)	3.33 (2.67, 4.33)	3.84 (3.00, 5.67)	U = 5229.5	0.005**	r = 0.19
3D driving errors, Mdn (IQR)	0 (0, 0.33)	0.17 (0, 0.67)	U = 4865.0	<0.001***	r = 0.25
Physical activity					
Strenuous exercise, Mdn (IQR)	3 (1, 4)	1 (0, 2)	U = 3648.0	<0.001***	r = 0.37
Moderate exercise, Mdn (IQR)	2 (1, 4)	2 (0, 3)	U = 4821.5	0.001**	r = 0.22
Mild exercise, Mdn (IQR)	5 (2, 7)	3 (1, 6)	U = 5421.5	0.039*	r = 0.14
Leisure time activity score, Mdn (IQR)	38 (23, 54.75)	15 (5, 34.75)	U = 3312.5	<0.001***	r = 0.42
Covariates					
Spatial activity ratio, Mdn (IQR)	0.86 (0.57, 1.11)	1.67 (1.10, 2.45)	U = 2421.0	<0.001***	r = 0.48
Sense of direction, M (SD)	4.93 (1.28)	4.06 (1.24)	t = 5.19	<0.001***	d = 0.69
Spatial anxiety, Mdn (IQR)	23 (17, 27)	20 (15, 25)	U = 5334.5	0.014*	r = 0.16

IQR, interquartile range; SD, standard deviation; Mdn, median; M, mean Effect sizes: r for Mann–Whitney U tests, Cohen’s d for *t*-tests *p < 0.05; **p < 0.01; ***p < 0.001.

### Visuospatial cognitive performance

Consistent with our first hypothesis, males significantly outperformed females across all visuospatial cognitive domains. Effect sizes ranged from medium to large (r = 0.19–0.34). In mental rotation tasks, males scored higher in both low difficulty (Mdn = 8 vs. 7, U = 5244.5, *p* = 0.004, r = 0.19) and high difficulty conditions (Mdn = 9 vs. 7.5, U = 4296.5, *p* < 0.001, r = 0.34), with larger sex differences emerging at higher difficulty levels.

For spatial memory, males demonstrated higher span products in both forward (Mdn = 406 vs. 288, U = 4290.0, *p* < 0.001, r = 0.34) and backward conditions (Mdn = 291 vs. 258, U = 4549.5, *p* < 0.001, r = 0.31). Notably, clicking velocity did not differ between sexes [forward: *t*(222) = 1.16, *p* = 0.249; backward: U = 6176.5, *p* = 0.899], suggesting that sex differences reflected memory capacity rather than motor speed.

In spatial navigation tasks, males completed mazes faster than females in both 10 × 10 (Mdn = 22 vs. 26.5 s, U = 4753.0, *p* < 0.001, r = 0.30) and 18 × 18 conditions (Mdn = 64.5 vs. 76.5 s, U = 5075.5, *p* = 0.002, r = 0.21). The number of steps did not differ significantly, confirming that performance differences reflected decision-making speed rather than path efficiency.

### Physical activity patterns

Males reported significantly higher levels of physical activity across all intensity categories. The most pronounced difference was in strenuous exercise (males: Mdn = 3, IQR = 1–4; females: Mdn = 1, IQR = 0–2; U = 3648.0, *p* < 0.001, r = 0.37). Consequently, males had substantially higher leisure time activity scores (Mdn = 38 vs. 15, U = 3312.5, *p* < 0.001, r = 0.42), representing a large effect size.

### Simple mediation analysis: leisure time activity score

**Figure 2:**
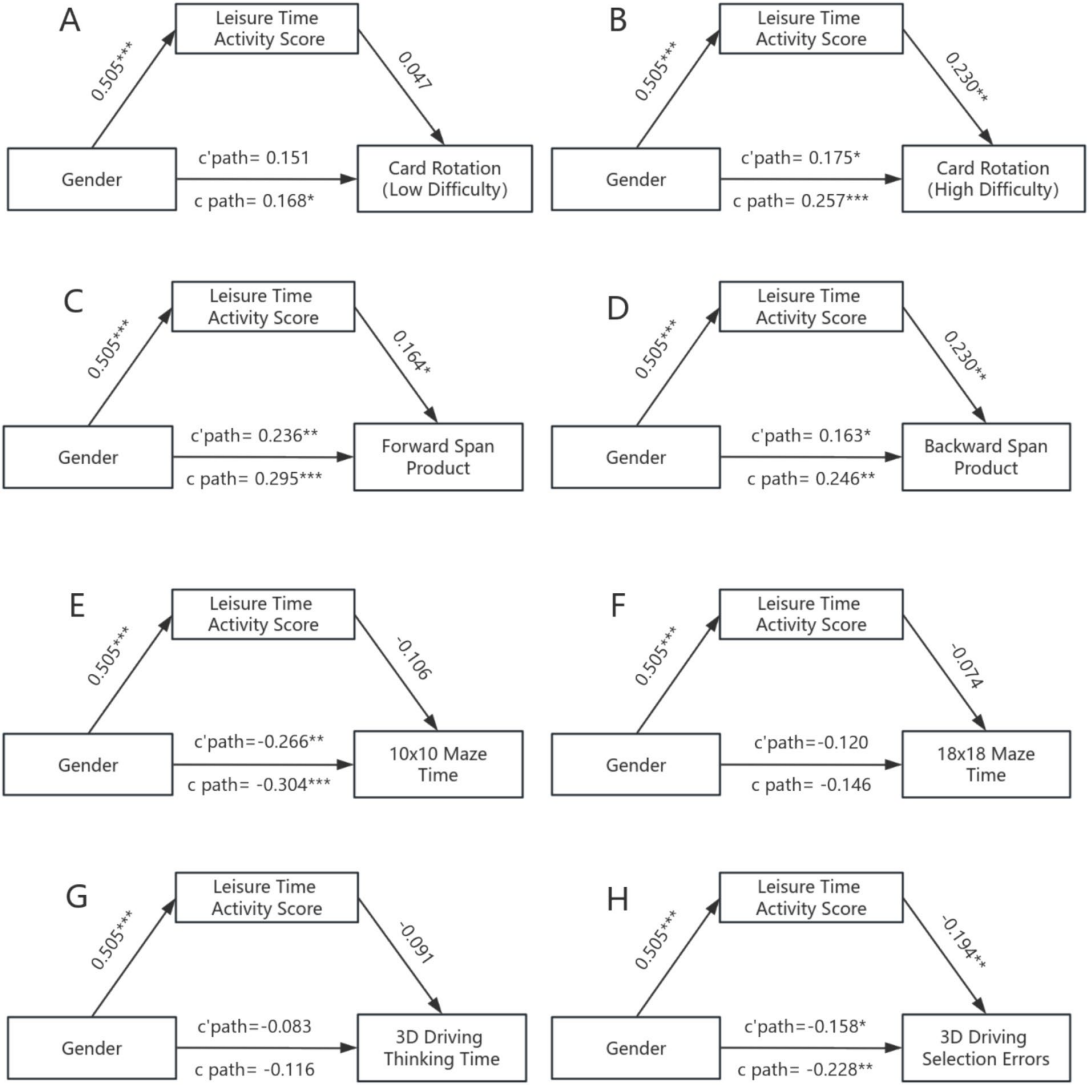
Simple mediation model: leisure time activity score as mediator of sex differences in visuospatial cognition. Standardized coefficients shown for mediation models examining leisure time activity score as a mediator between sex (0 = male, 1 = female) and visuospatial performance across eight cognitive outcomes. Numbers on paths represent standardized regression coefficients. c = total effect; c’ = direct effect after controlling for mediator; ab = indirect effect through mediator. All models controlled for spatial anxiety, sense of direction, and childhood spatial activity ratio. Significant indirect effects (*p* < 0.05) are indicated by asterisks. Results demonstrate partial mediation for mental rotation (high difficulty), spatial memory (forward and backward span products), and 3D driving errors, with proportion mediated ranging from 20 to 34%.

**Figure 3:**
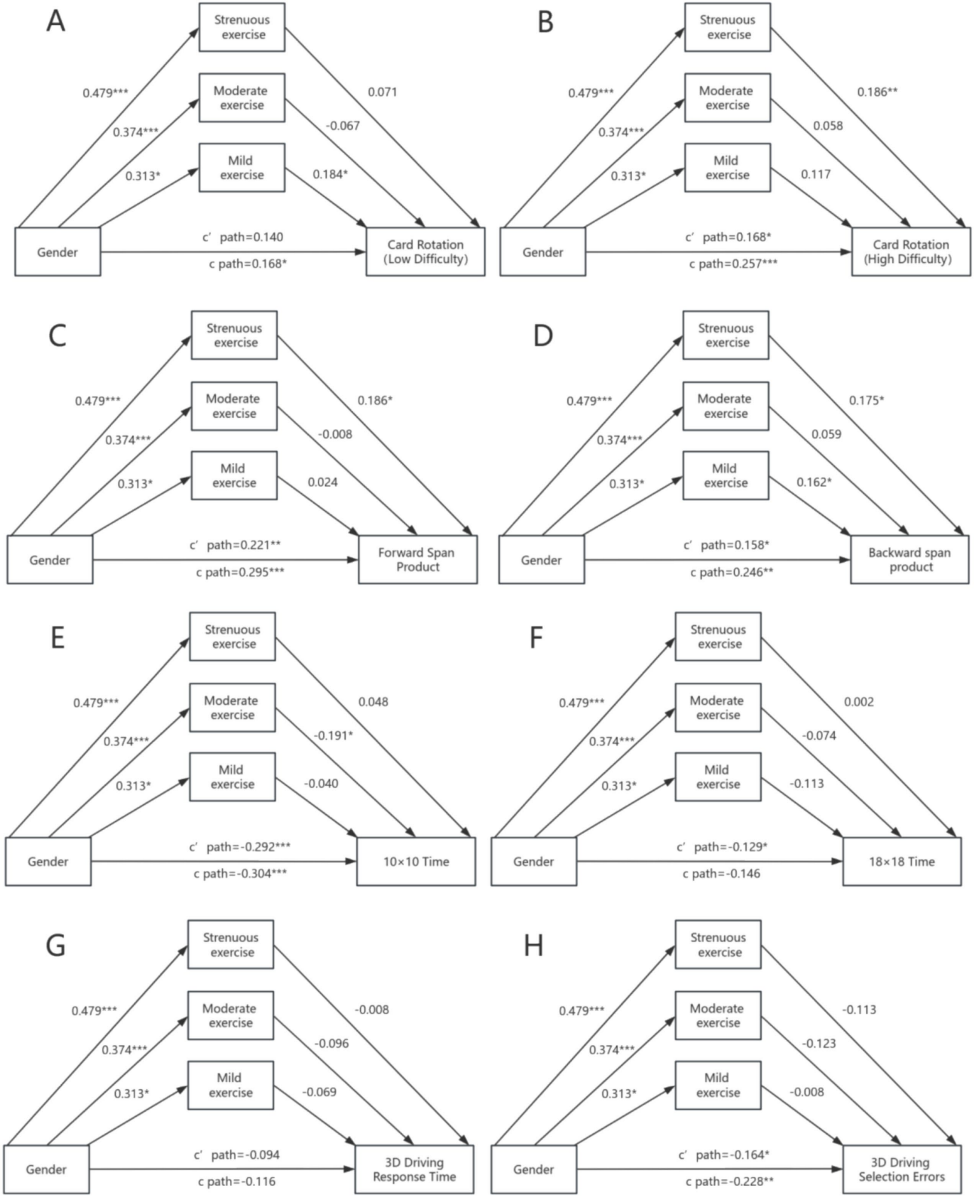
Parallel mediation model: exercise intensity types as mediators of sex differences in visuospatial cognition. Parallel mediation analysis with three exercise intensity levels (strenuous, moderate, mild) as simultaneous mediators between sex and visuospatial performance. Path coefficients represent standardized effects for each mediator controlling for others. Strenuous exercise (solid lines) emerged as the primary significant mediator for mental rotation (high difficulty), spatial memory (forward and backward spans), and 3D driving errors. Moderate exercise (dashed lines) showed limited mediation only for 10 × 10 maze navigation. Mild exercise (dotted lines) showed no significant mediation effects. All models controlled for spatial anxiety, sense of direction, and childhood spatial activity ratio. *p < 0.05; **p < 0.01; ***p < 0.001.

As shown in Figure 2 and Table 2, leisure time activity score significantly mediated sex differences in several visuospatial domains. The proportion mediated (PM) varied by task complexity, ranging from 10.1% for the low-difficulty card rotation to 34% for more demanding tasks (backward span). Significant indirect effects were observed for:

- Mental rotation (high difficulty): indirect effect = 0.083, 95% CI [0.029, 0.145], PM = 32% (Figure 2B)
- Forward spatial memory: indirect effect = 0.059, 95% CI [0.008, 0.125], PM = 20% (Figure 2C)
- Backward spatial memory: indirect effect = 0.083, 95% CI [0.029, 0.153], PM = 34% (Figure 2D)
- 3D driving errors: indirect effect = −0.070, 95% CI [−0.129, −0.024], PM = 31% (Figure 2H)

**Table 2:** Simple mediation analysis: leisure time activity score as mediator.

Outcome	Total effect (c)	Direct effect (c’)	Indirect effect (ab)	PM (%)	95% CI
Card rotation (low)	0.168 (0.099, 0.237)	0.151 (−0.015, 0.317)	0.017 (−0.033, 0.068)	10.1	[−0.033, 0.068]
Card rotation (high)**	0.257 (0.111, 0.404)	0.175 (0.023, 0.326)	0.083 (0.029, 0.145)*	32.3	[0.029, 0.145]
Forward span product**	0.295 (0.144, 0.445)	0.236 (0.078, 0.394)	0.059 (0.008, 0.125)*	20.0	[0.008, 0.125]
Backward span product**	0.246 (0.097, 0.395)	0.163 (0.009, 0.318)	0.083 (0.029, 0.153)*	33.7	[0.029, 0.153]
10 × 10 maze time	−0.304 (−0.457, −0.152)	−0.266 (−0.427, −0.105)	−0.038 (−0.083, 0.004)	12.5	[−0.083, 0.004]
18 × 18 maze time	−0.146 (−0.304, 0.012)	−0.120 (−0.287, 0.048)	−0.026 (−0.091, 0.029)	17.8	[−0.091, 0.029]
3D driving time	−0.116 (−0.268, 0.037)	−0.083 (−0.244, 0.078)	−0.033 (−0.085, 0.012)	28.4	[−0.085, 0.012]
3D driving errors**	−0.228 (−0.379, −0.077)	−0.158 (−0.316, −0.000)	−0.070 (−0.129, −0.024)*	30.7	[−0.129, −0.024]

Values in parentheses represent 95% confidence intervals. PM, Proportion mediated. All models controlled for spatial anxiety, sense of direction, and childhood spatial activity ratio. *Significant indirect effect (95% CI excludes zero); **significant after FDR correction. Note on PM range: the 20–34% range referenced in text reflects tasks showing significant mediation after FDR correction. The 10.1% value for Card Rotation (low) represents a non-significant trend (95% CI includes zero).

Importantly, direct effects of sex remained significant in all models, indicating partial rather than full mediation.

This pattern of results suggests a dose–response relationship between task complexity and exercise mediation effects. While strenuous exercise mediated 20–34% of sex differences in high-demand tasks (mental rotation-high, spatial memory, 3D navigation), its mediating role was attenuated (PM = 10.1%) for the low-difficulty card rotation task. This gradient effect aligns with neuroplasticity models positing that exercise benefits are most pronounced for tasks requiring executive control and mental transformation.

### Parallel mediation analysis: exercise intensity

To examine the differential mediating effects of exercise intensity, we conducted parallel mediation analyses with strenuous, moderate, and mild exercise as simultaneous mediators (Figure 3 and Table 3). Multicollinearity diagnostics indicated acceptable VIF values (range: 1.23–1.67) for all exercise variables.

**Table 3:** Parallel mediation analysis: exercise intensity as mediators.

Outcome	Total indirect	Strenuous exercise	Moderate exercise	Mild exercise
Card rotation (low)				
Effect (95% CI)	0.029 (−0.031, 0.088)	0.028 (−0.027, 0.084)	−0.010 (−0.046, 0.014)	0.011 (−0.018, 0.045)
PM (%)	17.3	16.7	−6.0	6.5
Card rotation (high)				
Effect (95% CI)	0.089 (0.032, 0.156)*	0.073 (0.021, 0.134)*	0.009 (−0.014, 0.037)	0.007 (−0.013, 0.035)
PM (%)	34.6	28.4	3.5	2.7
Forward span product				
Effect (95% CI)	0.073 (0.016, 0.150)*	0.073 (0.017, 0.147)*	−0.001 (−0.027, 0.030)	0.002 (−0.012, 0.020)
PM (%)	24.7	24.7	−0.3	0.7
Backward span product				
Effect (95% CI)	0.088 (0.024, 0.166)*	0.069 (0.009, 0.143)*	0.009 (−0.015, 0.041)	0.010 (−0.016, 0.040)
PM (%)	35.8	28.0	3.7	4.1
10 × 10 maze time				
Effect (95% CI)	−0.012 (−0.072, 0.047)	0.019 (−0.031, 0.076)	−0.029 (−0.071, −0.001)*	−0.002 (−0.020, 0.008)
PM (%)	3.9	−6.3	9.5	0.7
18 × 18 maze time				
Effect (95% CI)	−0.017 (−0.083, 0.048)	0.001 (−0.057, 0.062)	−0.011 (−0.048, 0.011)	−0.007 (−0.035, 0.012)
PM (%)	11.6	−0.7	7.5	4.8
3D driving time				
Effect (95% CI)	−0.022 (−0.077, 0.033)	−0.003 (−0.049, 0.045)	−0.014 (−0.044, 0.005)	−0.004 (−0.025, 0.010)
PM (%)	19.0	2.6	12.1	3.4
3D driving errors				
Effect (95% CI)	−0.064 (−0.123, −0.016)*	−0.045 (−0.092, −0.004)*	−0.019 (−0.052, 0.001)	−0.001 (−0.016, 0.015)
PM (%)	28.1	19.7	8.3	0.4

PM, Proportion mediated. All models controlled for spatial anxiety, sense of direction, and childhood spatial activity ratio. *Significant indirect effect (95% CI excludes zero).

Strenuous exercise emerged as the primary mediator across multiple domains:

*Mental rotation:* only in high difficulty tasks did strenuous exercise show significant mediation (indirect effect = 0.073, 95% CI [0.021, 0.134], PM = 28%). No significant mediation was observed for low difficulty tasks, supporting our hypothesis that mediation effects would be stronger for more cognitively demanding tasks (Figures 3A,B).

*Spatial memory*: strenuous exercise significantly mediated sex differences in both forward (indirect effect = 0.073, 95% CI [0.017, 0.147], PM = 25%) and backward span (indirect effect = 0.069, 95% CI [0.009, 0.143], PM = 28%). The slightly stronger effect for backward span aligns with its greater executive control demands (Figures 3C,D).

*Spatial Navigation*: An unexpected finding emerged for the 10 × 10 maze, where moderate exercise showed a small but significant mediating effect (indirect effect = −0.029, 95% CI [−0.071, −0.001], PM = 10%). Strenuous exercise did not mediate navigation performance (Figures 3E,F).

*3D driving test*: strenuous exercise mediated sex differences in selection errors (indirect effect = −0.045, 95% CI [−0.092, −0.004], PM = 20%) but not thinking time (Figures 3G,H).

### FDR correction and sensitivity analyses

After applying FDR correction for multiple comparisons, all originally significant indirect effects remained significant (adjusted *p* < 0.05). Sensitivity analyses excluding participants with extreme exercise scores (*n* = 8) yielded similar results, supporting the robustness of our findings.

## Discussion

This study provides novel evidence that physical exercise, particularly strenuous exercise, partially mediates sex differences in visuospatial cognitive abilities among young adults. Our findings contribute to understanding the multifactorial nature of cognitive sex differences and highlight the potential role of modifiable lifestyle factors in shaping cognitive abilities.

### Sex differences in visuospatial cognition

Our results replicate and extend previous findings of male advantages in visuospatial tasks (Voyer et al., 1995; Weigelt and Memmert, 2021). The observed effect sizes (r = 0.19–0.34) align with meta-analytic estimates, with the largest differences emerging in mental rotation and spatial memory tasks. Importantly, our use of untimed tests suggests that these differences reflect genuine cognitive abilities rather than processing speed advantages, addressing a longstanding methodological concern in the literature (Boone and Hegarty, 2017).

The pattern of results supports a nuanced view of sex differences in spatial cognition. While males showed advantages across all domains, the magnitude varied by task demands. The largest effects emerged in tasks requiring active mental manipulation (mental rotation) and spatial-sequential processing (spatial memory), while smaller effects were observed in navigation tasks. This pattern suggests that sex differences may be most pronounced in tasks requiring dynamic spatial transformations rather than static spatial knowledge.

### Exercise as a mediator

A key contribution of this study is demonstrating that physical exercise partially mediates sex differences in visuospatial abilities, accounting for 20–34% of the total effects. This finding extends previous research on exercise-cognition relationships (Rogge et al., 2017; Lin et al., 2022; Liu-Ambrose et al., 2021; Ludyga et al., 2022) by revealing exercise as a mechanism underlying cognitive sex differences.

The specificity of strenuous exercise as the primary mediator aligns with neurobiological evidence. The observed mediation gradient - from 10.1% in low-difficulty tasks to 34% in high-demand tasks - mirrors established dose–response curves in exercise-cognition research (Cassilhas et al., 2016). High-intensity exercise produces more robust increases in brain-derived neurotrophic factor (BDNF), insulin-like growth factor-1 (IGF-1), and vascular endothelial growth factor (VEGF) (Voyer et al., 1995; Weigelt and Memmert, 2021), all of which promote hippocampal neurogenesis and synaptic plasticity. Moreover, strenuous exercise induces greater acute elevations in sex hormones (Pinker, 1984), which may interact with exercise-induced neuroplasticity to enhance spatial cognition.

The stronger mediation effects for cognitively demanding tasks (high difficulty mental rotation, backward spatial span) support our hypothesis and suggest that exercise benefits may be most apparent when cognitive resources are taxed. This pattern parallels findings in aging research, where exercise effects are often strongest for executive-demanding tasks (Lin et al., 2022).

### Theoretical and practical implications

Our findings contribute to theoretical models of cognitive sex differences by identifying a modifiable behavioral factor that partially accounts for these differences. Traditional explanations have emphasized relatively fixed factors such as prenatal hormone exposure, brain structure, and early socialization (Caldera et al., 1989; Terlecki and Newcombe, 2005; Nazareth et al., 2013). While these factors remain important, our results suggest that contemporary lifestyle differences also contribute to maintaining cognitive sex differences into adulthood.

From a practical perspective, these findings suggest that encouraging greater participation in strenuous physical activities among women could help reduce gender disparities in spatial abilities. This has potential implications for STEM education and careers, where spatial abilities predict success. However, addressing the gender gap in physical activity requires understanding and removing barriers to women’s exercise participation, including social, cultural, and environmental factors (The Lancet Public H, 2019).

### Limitations

Several limitations warrant consideration. First, the cross-sectional design precludes causal inferences. While we conceptualized exercise as mediating the relationship between sex and spatial abilities, alternative causal pathways are plausible. Individuals with better spatial abilities might be more inclined to engage in sports requiring spatial skills, creating a bidirectional relationship. Longitudinal and intervention studies are needed to establish temporal precedence and causality.

Second, physical activity assessment relied on self-report, which may overestimate actual activity levels. The GSLTPAQ also captures only frequency, not duration or intensity within categories. Future research should incorporate objective measures (accelerometry) and more comprehensive assessment of exercise characteristics including type, duration, and context.

Third, while we controlled for several relevant covariates, unmeasured confounders may influence the observed relationships. Factors such as general cognitive ability, socioeconomic status, nutrition, sleep quality, and genetic predispositions could affect both exercise behavior and spatial cognition. Additionally, personality traits such as competitiveness or risk-taking might influence both exercise engagement and spatial task performance.

Fourth, our sample consisted of university students from a relatively homogeneous background. The restricted range of cognitive abilities and exercise levels in this population may underestimate true population effects. Replication in more diverse samples is needed to establish generalizability.

Finally, while we examined multiple aspects of visuospatial cognition, our battery did not include all spatial abilities. Future research should examine whether exercise mediation extends to other spatial domains such as spatial visualization, perspective-taking, and real-world navigation.

### Future research directions

Building on these findings, several research directions merit pursuit:

*Intervention studies*: randomized controlled trials examining whether exercise training reduces sex differences in spatial abilities would provide causal evidence. Such studies should compare different exercise types and intensities.

*Mechanistic research*: neuroimaging and biomarker studies could elucidate the neurobiological pathways linking exercise, sex, and spatial cognition. Key targets include hippocampal volume, white matter integrity, and neurotrophic factors.

*Developmental perspectives*: longitudinal studies tracking exercise patterns and spatial ability development from childhood through adulthood could identify critical periods for intervention.

*Cultural comparisons*: cross-cultural research could examine whether the mediating role of exercise varies across societies with different gender norms regarding physical activity.

## Conclusion

This study provides novel evidence that physical exercise partially mediates sex differences in visuospatial cognitive abilities among young adults, with strenuous exercise showing the strongest mediating effects. These findings extend our understanding of cognitive sex differences beyond traditional biological and sociocultural explanations to include modifiable lifestyle factors. While the cross-sectional design limits causal inferences, our results suggest that promoting increased physical activity, particularly strenuous exercise, among women may help reduce gender disparities in spatial cognition. Future longitudinal and intervention research is needed to confirm these relationships and explore their practical applications in educational and occupational settings.

## Data Availability

The original contributions presented in the study are included in the article/supplementary material, further inquiries can be directed to the corresponding author.
